# Assessment of Time in Therapeutic Range (TTR) in a Primary Care Warfarin Clinic

**DOI:** 10.7759/cureus.85653

**Published:** 2025-06-09

**Authors:** Divya Viswanathan, Abiram Sivanandam, Piyumika De Silva

**Affiliations:** 1 Internal Medicine, Rutgers University New Jersey Medical School, Newark, USA

**Keywords:** anticoagulation, atrial fibrillation, atrial fibrillation (af), bleeding, thrombosis, warfarin

## Abstract

Background

Anticoagulation is commonly used to prevent thromboembolic events. Warfarin is a cost-effective, widely used anticoagulant that requires close monitoring due to its narrow therapeutic index. Time in therapeutic range (TTR) is a measure of international normalized ratio (INR) control. Using a retrospective approach, we evaluated the TTR of patients using warfarin for anticoagulation in our state-owned urban primary care clinic.

Methods

We conducted a retrospective chart review of adult patients on warfarin therapy followed at an urban Level 1 trauma center’s ambulatory clinic from January to December 2024. Patients with at least two contiguous INR visits over a two-month period were included. Data on warfarin dosing, INR values, anticoagulation visit history, and demographics were extracted from the electronic medical record. The primary outcome was TTR, calculated using the traditional method. Statistical analysis was performed using R-studio.

Results

Overall, 103 patients were analyzed in our clinic. The average TTR was 46.7% for this population. Around 17.5% of patients had a TTR >70%, and 30.1% had a TTR >60%. Approximately 29.1% of patients presented with high bleeding risk (INR >4.5) at least once during the measured time period, with 5.8% of patients requiring ED visits for significant elevations in INR. Variables such as gender, age, and insurance status did not significantly contribute to measured TTR.

Conclusions

The average TTR of our patient population was suboptimal. These findings highlight the need for more targeted quality improvement efforts to enhance anticoagulation management in our primary care clinic. Further evaluation of our current protocol with the help of pharmacists and the enhancement of patient education may be beneficial in achieving this goal.

## Introduction

Anticoagulant medication is used to control, treat, and prevent thromboembolic events in patients at high risk for such events. This population includes patients with a history of atrial fibrillation, deep vein thrombosis (DVT), pulmonary embolism, and mechanical valves. Warfarin is a widely prescribed anticoagulant [1], particularly for its cost-effectiveness. Warfarin exhibits anticoagulation properties by inhibiting vitamin K epoxide reductase [1], decreasing total body vitamin K available for synthesis of clotting factors II, VII, IX, and X, which are dependent on vitamin K for formation. 

An international normalized ratio (INR) of 2.0-3.0 has been recognized as a therapeutic laboratory range for warfarin dosing for a majority of indications, with some exceptions requiring an INR of 2.5-3.5, such as mitral valve replacements or multiple risk factors for thrombosis [1]. INR is calculated by using a patient’s prothrombin time (PT) divided by a standardized control PT established by the WHO [2]. Although effective in preventing thromboembolism, warfarin is notable for its narrow therapeutic index, variable dosing requirements, and interactions with other common medications. Notably, an INR greater than or equal to 4.5 has been associated with increased risk of bleeding in these patients [3]. Factors such as ingestion of vitamin K-rich foods, age, liver dysfunction, infection, and genetic polymorphisms in the cytochrome system affect warfarin dosing required to achieve a therapeutic range [4]. Due to these factors, patients are required to obtain blood work and attend medical visits frequently for medication adjustments. 

The effectiveness of INR control is most commonly measured by assessing the percentage of time in therapeutic range (TTR) [5]. Generally, an individual TTR greater than 70% indicates that a patient is well maintained on their warfarin regimen [6].

There are three methods that have been used to calculate TTR. In the traditional method, TTR is computed by calculating the ratio of visits where INR is within goal to the total number of visits. This is a simple and easy-to-compute method [7]. Its disadvantages include a lack of account for the time between INR measurements and possible over- or underestimation of TTR in patients with infrequent INR testing. Another method used is the Rosendaal method, which estimates TTR by assuming a linear relation between two INR values and assigning an estimated INR to each day between test values. Because of this assumption, this method may not accurately reflect true variation in populations with variable adherence or follow-up and may be affected by extremely out-of-range values [8]. The cross-sectional method is used to calculate TTR by taking all patients' INR values on the same date and calculating the proportion of patients within the therapeutic range. This method uses one value per patient and does not reflect longitudinal control of each patient [8].

The main objective of this study is to evaluate the TTR, and by extension INR control, in patients receiving warfarin anticoagulation managed by internal medicine residents at an urban primary care clinic. Our main objective is to analyze the number of patients who are achieving adequate anticoagulation (TTR ≥70%), analyze potential demographic characteristics associated with subtherapeutic INR control, and quantify the number of patients without guideline-supported indications for warfarin. Our study also seeks to identify points of improvement within our current INR monitoring protocol and make modifications to this protocol based on the results of this study. 

## Materials and methods

The subject population was characterized as patients on active warfarin therapy attending visits at a large urban Level 1 trauma center’s ambulatory clinic within the Northeast United States from January 2024 to December 2024. The inclusion criteria for this study were patients who had been prescribed warfarin therapy and had attended INR checks at our ambulatory care clinic. Exclusion criteria included patients who attended less than two visits and those who did not participate in anticoagulation visits continuously for at least two months. A retrospective cohort analysis was obtained through the Epic electronic medical record (EMR) system. Information such as daily warfarin dosage, INR laboratory values, indication for anticoagulation, scheduled anticoagulation, and visit information was collected. Demographic data, including age, race, gender, and insurance status, were collected through the Epic EMR system and used to stratify the patients into subgroups. 

**Figure 1:**
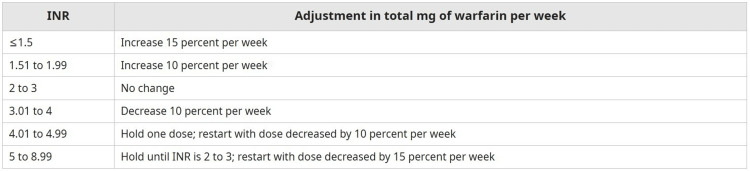
Warfarin dosing adjustment guidelines INR, international normalized ratio

Our current clinic protocol involves adjusting warfarin dosing based on protocols in UpToDate (Figure 1). Patients with INR in goal for one week are scheduled to follow up in one week, those in goal for two weeks have follow up in two weeks, those in goal for three weeks are scheduled for follow up in three weeks, and any patient in goal for longer than four or more weeks is scheduled for follow up in four weeks. During the visit, patients are asked about changes in alcohol use, tobacco use, eating habits, missed doses, scheduled procedures, and potential symptoms of bleeding or thrombosis. Patients then have their warfarin dose adjusted and are scheduled for a follow-up visit. 

The primary outcome studied was TTR for all patients within the study population. The traditional method was used to calculate TTR for all patients over this time period, which reports the proportion of INR values within range per patient. This approach was selected for its simplicity and alignment with our clinic's data, where INR testing is often performed infrequently or at variable intervals. The traditional method avoids assumptions about INR stability between tests and uses multiple INR values, which are important factors in a population with inconsistent follow-up periods. This methodology is also not heavily affected by INR values that may be significantly out of range. Mann-Whitney U tests were used to evaluate differences in TTR based on race, insurance status, and age groups. Kruskal-Wallis rank sum tests were used to assess differences based on gender and warfarin dosing and identify significant differences in TTR within sample subgroups. Our study was exempt from the Institutional Review Board as it was a quality improvement initiative based on retrospective chart review. 

## Results

Overall and demographic analyses

A total of 103 patients, 59 male and 44 female, were identified in this population with a median age of 58 years. The population contained Black (N=43), Hispanic (N=52), White (N=5), and other (N=3) patients. A majority of patients had no insurance (N=31) or charity care (N=23); a financial assistance insurance program was provided by hospitals to low-income or uninsured individuals. 

**Figure 2:**
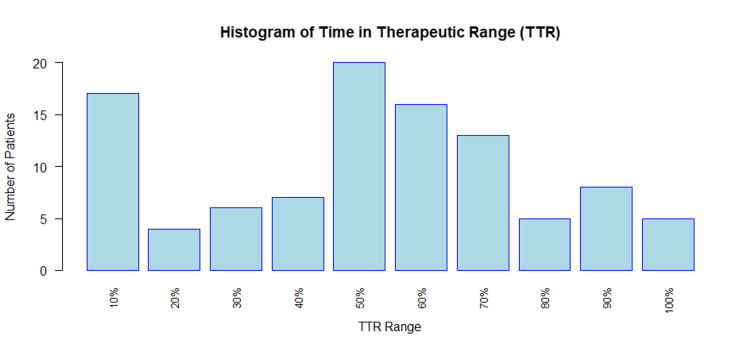
Histogram of time in therapeutic range of warfarin clinic patients

The overall TTR for this patient population was suboptimal. The average TTR for the population (N=103) was 46.7%. When evaluating effective anticoagulation, 18 (17.48%) patients had a TTR >70%, and 31 (30.10%) patients had a TTR >60%. These values were further quantified based on TTR in Figure 2.

Race appeared to be the only demographic factor with a statistically significant difference in TTR in this population. TTR was not statistically different between males and females (p=0.96). White patients had statistically significantly higher TTR (61.70%) than Black (46.47%, p=0.05) or Hispanic patients (44.94%, p=0.04). There was no statistically significant difference in TTR between insurance status types (p=0.90). Patients aged 50-60 had the highest TTR of 52%, although it was not significantly higher than any other age group (p=0.69). Further demographic characteristics and corresponding TTR values are displayed in Table 1.

**Table 1:** Demographic characteristics and average TTR of patients on warfarin anticoagulation INR, international normalized ratio; TTR, time in therapeutic range

Characteristic	Count (N)	Average TTR (%)
Female	44	46.51
Male	59	46.82
Race	N/A	N/A
Black	43	46.47
White	5	61.70
Hispanic	52	44.94
Other	3	53.85
INR ranges	N/A	N/A
2.0-2.5	1	25.00
2.0-3.0	80	45.08
2.5-3.0	1	47.83
2.5-3.5	21	53.62
Insurance status	N/A	N/A
Private insurance	13	49.86
Medicaid	17	40.80
Medicare	19	47.30
Charity care	23	46.04
None	31	48.56

Indications for anticoagulation

The most common documented reason for warfarin anticoagulation was atrial fibrillation. Only 40 patients indicated to be on anticoagulation with warfarin as a first-line agent, most commonly due to mechanical valve replacements. This is further detailed in Table 2. Of the patients not indicated to be on warfarin as the first line, eight patients were switched to apixaban, two to rivaroxaban, and two to enoxaparin. Overall, five patients discontinued warfarin, with three patients completing their course of anticoagulation (for DVT), one patient converting to hospice, and one case in which anticoagulation was not indicated. 

**Table 2:** Indications for warfarin anticoagulation AVR, aortic valve replacement; CVA, cerebrovascular accident; IVC, inferior vena cava; LA, left atrial; LV, left ventricular; MVR, mitral valve replacement

Characteristic	Count (N)	Percentage (%)
Multiple indications, warfarin not indicated	26	25.24
Multiple indications, warfarin indicated	16	15.53
Atrial fibrillation	13	12.62
Mechanical AVR	12	11.65
Deep vein thrombosis	8	7.77
Mechanical MVR	8	7.77
Antiphospholipid syndrome	5	4.85
LV thrombus	3	2.91
Pulmonary embolism	3	2.91
IVC thrombus	2	1.94
Atrial flutter	1	0.97
Cerebral thrombosis	1	0.97
LA thrombus	1	0.97
Lambl's excrescence of aortic valve	1	0.97
Portal vein thrombosis	1	0.97
Protein S deficiency	1	0.97
Secondary CVA prevention	1	0.97

Evaluating average warfarin dose 

The average daily warfarin dose for the cohort was 7 mg. There were 61 patients with a daily warfarin dose less than 7 mg, and 42 patients with a daily warfarin dose greater than or equal to 7 mg. Patients with an average daily warfarin dose greater than 7 mg had an average TTR of 48.80%, whereas those taking less than 7 mg had a lower TTR of 45.05% (p=0.51). Patients with an INR goal of 2.0-3.0 had an average daily warfarin dosage of 7.14 mg (5.86-7.74, 95% CI) and an average TTR of 44.22%. Those with an INR goal of 2.5-3.5 had an average daily warfarin dosage of 6.80 mg (5.79-8.50, 95% CI). Between patients with an INR goal of 2.0-3.0 and 2.5-3.5, there was no statistically significant difference in average daily warfarin dose or TTR (p=0.48, p=0.38, respectively). 

Adverse events and missed visits

Several patients had elevated INR values, leading to an increased risk of bleeding. Notably, 30 patients had an INR measurement greater than 4.5 at least once throughout the calendar year. The median number of visits with an INR >4.5 was one; the average daily warfarin dose was 6.56 mg, and the average TTR was 46.18%. Of these patients, six were sent to the ED. One of these patients was hospitalized for gastrointestinal bleeding. Patients sent to the ED had an average TTR of 38.15% and an average daily warfarin dose of 7 mg.

There appeared to be a number of missed visits, leading to limitations on proper dose adjustments in this population. Patients were scheduled on average to have 18 visits yearly and missed about four of these visits. There was no strong correlation between the number of missed visits and TTR (correlation coefficient = 0.076). In this cohort, 68% of patients had more visits with an INR below goal than above. Patients had, on average, four visits with an INR below goal and two visits with an INR above goal per year. 

## Discussion

TTR is a recognized metric for the performance of oral anticoagulation. Effective INR control has been cited as ranging from 60%-70% based on various guidelines [9,10]. The most recent CHEST guidelines from 2018 recommend interventions for TTR <65%, including more frequent INR visits, reviewing medications, and further education with counseling [11]. This study showed that patients receiving care in an ambulatory care center had an average TTR of 46.70%. A similar study assessing TTR of 5,210 patients with atrial fibrillation on warfarin therapy in the United States showed an average TTR of 65% [12]. Our study differs in that it included patients with a variety of indications for anticoagulation, including atrial fibrillation, mechanical valves, and recurrent DVT. 

Proposed areas of further investigation

A majority of the patients in our clinic did not have an indication for warfarin as first-line anticoagulation. Given that many of our patients were uninsured or had charity care, it is likely that cost played a role in the use of warfarin for first-line anticoagulation compared to direct oral anticoagulants or heparin-based products. Despite these findings, insurance status statistically did not have a difference in TTR. Thus, there may be other factors, alongside the cost of medication, which should be considered. Of those who did have an indication, prior mechanical valve replacement was the most common diagnosis. Interestingly, the average age of patients receiving valve replacements has been reported to be greater than 70 years old [13], whereas our patient population had a median age of 58, indicating that many of our patients have significant medical comorbidities necessitating such interventions. 

Our clinic is a primary care center with a majority of patients who are under charity care or are uninsured. Several social determinants of health may affect TTR and missed visits in our patient population. A majority of anticoagulation visits are scheduled as electronic health visits that require translator services. It is possible that language barriers may lead to miscommunication in anticoagulation instructions, information, and counseling. A study conducted at Massachusetts General Hospital showed that patients with limited English proficiency had significantly lower TTR values than those with English proficiency [14]. Further studies to address this in our population may be warranted. 

The patients receiving anticoagulation management at our clinic see multiple resident providers throughout the year who make changes to their warfarin dosing based on our standardized protocol. A study at John's Hopkin's found that TTR significantly increased in their patient population when one provider was primarily managing changes in warfarin dosing [15]. Adjusting our clinic scheduling to assign warfarin patients to a small, consistent group of residents may help improve TTR by enhancing continuity of care.

 Previous studies have shown that frequent fluctuations in a patient’s INR have been attributed to changes in dietary vitamin K intake [16]. A thorough review of dietary intake, alongside other relevant factors, by a physician and nutritionist would be beneficial in improving TTR in this population. Social factors such as access to technology and to transport in order to receive blood work may contribute to out-of-range TTR values. However, these challenges are inferred in our population, and further studies may be warranted to quantify the number of patients affected by such limitations. Prior studies have shown that psychosocial factors such as cognitive impairment, limited literacy, and social isolation may contribute to suboptimal TTR values [17]. There are limited prior studies evaluating the use of social workers to identify these barriers, but their support may be used to improve understanding and lead to further optimization of anticoagulation in our clinic. 

Enhancing patient education such as providing pamphlets or videos explaining the importance of warfarin compliance may aid in improving health literacy in our population. Educational interventions have been shown to improve TTR by explaining the risks and benefits of therapy, and interactions with other foods, tobacco, and alcohol [18]. Patients were then made to complete a worksheet-based exercise to consolidate their understanding from video-based education materials. 

Our current protocol may be changed to enhance patient education. Providing educational materials to all patients on warfarin to enhance understanding of the importance of anticoagulation and possible medication interactions. Referring patients to a nutritionist and having a social worker meet with patients may help identify dietary changes or social factors that may cause patients to need closer INR monitoring follow-up. Having patients scheduled with the same primary care resident provider may also aid in improving TTR to ensure continuity and understanding of the social factors that may affect INR levels. 

Visit frequency and cost considerations

The total number of scheduled INR check visits in our clinic was 1,877, with an average of 18 scheduled visits per patient. The total number of missed visits was 375, with an average of four missed visits per patient. A majority of our clinic's scheduled INR visits are telehealth visits. The number of scheduled INR checks and missed visits contributes significantly to the cost of our healthcare system and is further burdensome to our patients, as the average cost of an electronic health visit has been approximately $160 for an uninsured individual [19]. Improvement in TTR reduces the frequency of blood draws, office visits, and dosing changes, reducing the overall financial and time burden for our patients.

Limitations

It should be noted that there are several limitations to our study, particularly in the acquisition of data that may affect INR and warfarin dosing, such as the overall sample size of our patient population and subgroup disparities with mostly Hispanic and Black patients in our population. Our study did not analyze alcohol and tobacco use in this population. Tobacco use has been seen to lower INR levels due to enhanced warfarin clearance in smokers [20]. Chronic alcohol use has been seen to induce the CYP450 system [21], increasing warfarin metabolism, whereas acute alcohol use inhibits its metabolism. Increased alcohol consumption has also been associated with suboptimal TTR [22]. Further analysis of the use of these substances in the population would be beneficial in further understanding the potential root causes of fluctuations in INR. Furthermore, patients visiting our clinic for warfarin management did not have other comorbidities addressed at the time of visit. Thus, it is difficult to determine if their other medical conditions may have influenced TTR. Social limitations in our patient population (income, health literacy, and access to technology) were inferred and not directly measured.

## Conclusions

The overall TTR for this population was 46.7%, significantly lower than the accepted 60%-70% for adequate anticoagulation using warfarin. Demographic factors such as age, sex, insurance status, and missed visits were statistically not associated with lower TTR in our population, indicating that other factors may affect TTR in these patients. A multidisciplinary team, including primary care providers, social workers, nutritionists, and pharmacists, may be implemented in our clinic to aid in further identification of these barriers.
